# C-reactive protein as an early marker of sepsis resolution: results from the Portuguese Community-acquired Sepsis Study (SACiUCI study)

**DOI:** 10.1186/cc9692

**Published:** 2011-03-11

**Authors:** P Povoa, A Teixeira-Pinto, A Carneiro

**Affiliations:** 1 Hospital Sao Francisco Xavier, CHLO, Lisboa, Portugal; 2 Faculty of Medicine, University of Porto, Portugal; 3 Hospital Santo Antonio, Porto, Portugal

## Introduction

To assess the value of C-reactive protein (CRP) after prescription of antibiotics in order to define clinical resolution of community-acquired sepsis (CAS) admitted to the ICU.

## Methods

During 12 months a cohort multiple-centre observational study was conducted in 17 Portuguese ICUs segregating adults with CAS consecutively admitted. Patients were followed-up during the first 5 ICU days, the day of ICU discharge or death and hospital outcome. Comparison between survivors and nonsurvivors was performed.

## Results

Eight hundred and ninety-one patients (age 60 ± 17 years, hospital mortality 38%) were studied. At D1, CRP of survivors and nonsurvivors was not statistically different, 19.8 ± 12.5 mg/dl vs. 20.7 ± 12.8 mg/dl (*P *= 0.367). When we compared CRP of survivors and nonsurvivors at the different time points, we found that CRP of nonsurvivors was significantly higher since D3 onwards (*P *< 0.001, for D3, D4 and D5). After adjusting for SAPS II and severity of sepsis (sepsis, severe sepsis and septic shock), the initial value of CRP was notsignificantly associated with hospital mortality (OR_initial _= 1.01, 95% CI = (0.99, 1.02), *P *= 0.297). On the other side, the course of CRP, measuredas the relative change, obtained from a patient's specific linear model for the 5-day measurement of CRP generated two new variables, an intercept (describes the initial CRP value) and a slope (describes the CPR rate of change per day for a specific patient). We found that the slope was significantly associated with hospital mortality (OR CPR ratio = 1.03, 95% CI = (1.02, 1.04), *P *< 0.001). A patient with an average decrease of the CRP concentration of 10% per day has 32% less chance of dying when compared with a patient with the same SAPS II and the same severity of sepsis but with no decrease of the CRP. The area under the ROC curve for the model including SAPS II, severity of sepsis, initial CRP and CRP course, was 0.77. No significant differences between survivors and nonsurvivors were found on daily monitoring of temperature and white cell count, both at the first day (*P *= 0.799 and *P *= 0.496, respectively) and in the course of subsequent days (*P *= 0.360 and *P *= 0.594, respectively).

## Conclusions

Daily CRP measurement after antibiotic prescription was useful in identification, as early as day 3, of CAS patients with poor outcome. The slope of CRP course was markedly associated with prognosis.
